# Legal Restrictions on Gifts to Charity

**Published:** 1891-02-14

**Authors:** 


### Passing Topics.

### LEGAL RESTRICTIONS ON GIFTS TO CHARITY.

The paper under this title read before the Hospitals' Association by Mr. L. S. Bristowe, apparently was not considered attractive enough to ensure a full attendance of members. Nevertheless, both the paper and the discussion which followed proved not unworthy of the subject, important though it be. Everybody interested in our charities might well spare time to study this pamphlet if only to learn something of one of the difficulties under which charitable work is carried on in this country.

No doubt the Statute of Mortmain has answered some useful purpose, and at the time it was enacted very definite objects were sought to be attained by its means. But at present we are disposed to regard the Act as an anachronism. Institutions which relieve the State of onerous duties deserve well of the nation, and should not be subjected to uncalled-for hindrances. At the same time, the danger a speaker incurs by letting a good argument run away with him was well illustrated in the remarks of Mr. Amherst D. Tyssen, who essayed to maintain that if it be a public benefit to have charitable institutions, they should be supported out of the rates, a conclusion which drew forth from Mr. Burdett a natural and vigorous protest.

We think that managers of charitable institutions generally would be well satisfied to be allowed to live free from taxation, and would much prefer to forego more positive assistance—most certainly rate-rendered assistance. Whether in the case of organizations self-charged with so momentous a work as that our voluntary hospitals perform, the State should be content to leave everything to unaided private benevolence, and especially if private benevolence prove unequal to the task, is another and a wide question. But of this there ought to be no doubt —that the carrying out of the legitimate wishes of those who dispose of their property by will in aid of useful institutions should not be interfered with without grave reason. It is in this connection that we think the greatest objection must be found to the operation of the Act of Mortmain. It is now possible for the provisions of the statute to be strained by unscrupulous administrators in their own interests, and to the detriment of charities, and instances have occurred within our own knowledge where the intentions of testators have been frustrated in order that residuary legatees and others might benefit.

There is a particularly interesting and significant page in Mr. Bristowe's paper, where he shows the bewildering want of uniformity in regard to the decisions of the Courts. Thus the bonds of Dewsbury, Bradford, and Wakefield have been held not to be interests in land while a diametically opposite decision has been arrived at in the case of Salford and Oldham. Can the force of absurdity further go? Mr. Burdett said, and without doubt truly, that it is questionable whether many people are able and anxious to bequeath land to charities, but, however this may be, it is certain that there are multitudes of securities created of recent years which—themselves or their proceeds—might be, and often are bequeathed by testators in absolute ignorance of the fact that were the question raised they would be adjudged "impure personalty," and as such incapable of bequest to charities. This appears a very serious matter and even though the main principle of the Act of Mortmain be maintained, there seems abundant room for its modification and amendment in detail.

Agricola.

